# Expression Patterns of Key Hormones Related to Pea (*Pisum sativum* L.) Embryo Physiological Maturity Shift in Response to Accelerated Growth Conditions

**DOI:** 10.3389/fpls.2019.01154

**Published:** 2019-09-27

**Authors:** Federico M. Ribalta, Maria Pazos-Navarro, Kylie Edwards, John J. Ross, Janine S. Croser, Sergio J. Ochatt

**Affiliations:** ^1^Centre for Plant Genetics and Breeding, School of Agriculture and Environment, The University of Western Australia, Crawley, WA, Australia; ^2^School of Biological Sciences, University of Tasmania, Hobart, TAS, Australia; ^3^Agroécologie, AgroSup Dijon, INRA, Univ. Bourgogne Franche-Comté, Dijon, France

**Keywords:** abscisic acid, auxins, embryo physiological maturity, generation turnover, gibberellins, hormone regulation, legumes, precocious seed germination

## Abstract

Protocols have been proposed for rapid generation turnover of temperate legumes under conditions optimized for day-length, temperature, and light spectra. These conditions act to compress time to flowering and seed development across genotypes. In pea, we have previously demonstrated that embryos do not efficiently germinate without exogenous hormones until physiological maturity is reached at 18 days after pollination (DAP). Sugar metabolism and moisture content have been implicated in the modulation of embryo maturity. However, the role of hormones in regulating seed development is poorly described in legumes. To address this gap, we characterized hormonal profiles (IAA, chlorinated auxin [4-Cl-IAA], GA_20_, GA_1_, and abscisic acid [ABA]) of developing seeds (10–22 DAP) from diverse pea genotypes grown under intensive conditions optimized for rapid generation turnover and compared them to profiles of equivalent samples from glasshouse conditions. Growing plants under intensive conditions altered the seed hormone content by advancing the auxin, gibberellins (GAs) and ABA profiles by 4 to 8 days, compared with the glasshouse control. Additionally, we observed a synchronization of the auxin profiles across genotypes. Under intensive conditions, auxin peaks were observed at 10 to 12 DAP and GA_20_ peaks at 10 to 16 DAP, indicative of the end of embryo morphogenesis and initiation of seed desiccation. GA_1_ was detected only in seeds harvested in the glasshouse. These results were associated with an acceleration of embryo physiological maturity by up to 4 days in the intensive environment. We propose auxin and GA profiles as reliable indicators of seed maturation. The biological relevance of these hormonal fluctuations to the attainment of physiological maturity, in particular the role of ABA and GA, was investigated through the study of precocious *in vitro* germination of seeds 12 to 22 DAP, with and without exogenous hormones. The extent of sensitivity of developing seeds to exogenous ABA was strongly genotype-dependent. Concentrations between 5 and 10 µM inhibited germination of seeds 18 DAP. Germination of seeds 12 DAP was enhanced 2.5- to 3-fold with the addition of 125 µM GA_3_. This study provides further insights into the hormonal regulation of seed development and *in vitro* precocious germination in legumes and contributes to the design of efficient and reproducible biotechnological tools for rapid genetic gain.

## Introduction

Recent advances in LED light technology have enabled the development of protocols for rapid generation turnover of temperate legumes under conditions optimized for day-length, temperature, and light spectra (recently reviewed by Croser et al., 2018). These conditions act to compress time to flowering and seed development across diverse genotypes, but their effect on the hormone profile of developing embryos remains unknown. In pea (*Pisum sativum* L.), we have demonstrated that embryos do not efficiently germinate until maturity is reached at *c*. 18 days after pollination (DAP; Ribalta et al., 2017). However, application of exogenous hormones under *in vitro* culture conditions can lead to germination of immature embryos 10 to 12 DAP (Gallardo et al., 2006; Ochatt, 2011). Sugar metabolism and moisture content have been implicated in the modulation of embryo physiological maturity (Obendorf and Wettlaufer, 1984; Le Deunff and Rachidian, 1988; Weber et al., 2005). At 18 DAP, pea germinates when seed moisture content is below 60% and sucrose level is less than 100 mg g^−1^ dry weight (DW) (Ribalta et al., 2017). While sucrose and moisture are good indicators of readiness to germinate, questions remain about the hormonal regulation of the embryo maturation process in pea, particularly the role of abscisic acid (ABA) and gibberellins (GAs). We reason that exposure to intensive conditions optimized for rapid generation turnover will alter the hormone content and relationships within the developing seed, compressing the time to physiological maturity of the embryo.

Abscisic acid and GAs are well-known key regulators of seed maturation, dormancy, and germination (Finch-Savage and Leubner-Metzger, 2006). Abscisic acid mediates plant response to environmental conditions (Weber et al., 2005; Nakashima and Yamaguchi-Shinozaki, 2013) and is involved in the inhibition of precocious germination, in reserve mobilization (Bewley, 1997; Raz et al., 2001), and the regulation of mRNA transcription for storage proteins (Mc Carty, 1995; Bewley, 1997; Verdier et al., 2008; Ochatt, 2011). Abscisic acid biosynthesis takes place in both maternal and embryo tissues during seed maturation (Weber et al., 2005). Maternal ABA, synthesized in the seed coat of *Arabidopsis* and *Nicotiana* and translocated to the embryo, promotes seed growth and prevents abortion (Frey et al., 2004). In *Medicago truncatula* Gaertn., it has been suggested that ABA regulates germination through the control of radicle emergence by inhibiting cell-wall loosening and expansion (Gimeno-Gilles et al., 2009). In addition, ABA has been implicated in the regulation of starch biosynthesis and degradation pathways of developing seeds (Seiler et al., 2011). Gibberellins are known antagonists of ABA function in seed development and act primarily to promote germination-associated processes and seedling growth (Swain et al., 1995; Lee et al., 2002). Bioactive GAs (GA_1_, GA_4_, and GA_7_) are involved in determining the rate of seed coat growth and sink strength during the early stages of seed development (Nadeau et al., 2011). From 8 to 12 DAP, a transition in the seed GA biosynthesis and catabolism pathways occurs to produce sufficient bioactive GA for continued seed tissue growth and development, with a shift to the production of GA_20_ (precursor of GA_1_) and minimal bioactive GA in the embryo as the seed enters into its maturation phase (Ozga et al., 2009). In *Arabidopsis*, optimal germination requires the induction of GA biosynthesis to counteract the negative regulation imposed by DELLA proteins (Locascio et al., 2013; Resentini et al., 2015). Auxins play a key role during the early stages of seed development in processes such as cell division and elongation, nutrient accumulation, and water uptake (Pless et al., 1984; Vernoux et al., 2011; Ochatt, 2011; Atif et al., 2013). The “chlorinated auxin (4-Cl-IAA), a hormone restricted to the *Fabaceae* but not present in *Cicer* species (Lam et al., 2015), is thought to have a growth regulatory role in pea through the induction of GA biosynthesis and inhibition of ethylene action (Johnstone et al., 2005; Ozga et al., 2009). Hormone levels have been shown to substantially fluctuate according to the stage of seed development (Weber et al., 2005; Slater et al., 2013; Ochatt, 2015) and environmental conditions (Seiler et al., 2011; Yuan et al., 2011; Shu et al., 2016), although the influence of these changes on germination competence in legumes remains unclear.

In recent years, *in vitro* techniques have facilitated the study of the fundamental physiological mechanisms underlying seed development and germination (Le et al., 2010; Finkelstein, 2013; Ochatt, 2015; Gatti et al., 2016). Examples include studies of the kinetics of seed protein accumulation (Gallardo et al., 2006; Verdier et al., 2008), acquisition of stress tolerance (Elmaghrabi et al., 2018), and morphogenesis (Ochatt, 2011; Ochatt, 2013; Atif et al., 2013; Ribalta et al., 2017), as well as flowering and fruiting induced *in vitro* (Ochatt and Sangwan, 2008; Ochatt, 2011; Ribalta et al., 2014; Mobini et al., 2015). The use of plant growth regulators *in vitro* has also been explored as a means to elucidate hormonal regulation during embryo development in a number of species (Myers et al., 1990; Jimenez, 2005; Zhao et al., 2011; Abe et al., 2014), including legumes (Ozcan et al., 1993; Lakshmanan and Taji, 2000; Blöchl et al., 2005; Ochatt, 2011; Atif et al., 2013; Pazos-Navarro et al., 2017; Ochatt et al., 2018). Slater et al. (2013) studied the seed hormone profiles of developing *in vivo* seeds of four legume species in an effort to determine the optimal time for embryo rescue, although these predictions were not validated. Despite these efforts, little is known about the interactions between auxins, ABA, and GAs on the control of seed precocious *in vitro* germination in legumes.

In this research, we report hormonal profiles (IAA, 4-Cl-IAA, GA_20_, GA_1_, and ABA) of developing seeds at 10 to 22 DAP from phenologically diverse pea genotypes grown under intensive conditions optimized for rapid generation turnover and compare these profiles to those of equivalent samples from glasshouse conditions. To elucidate the biological relevance of these hormonal fluctuations to attainment of physiological maturity, in particular the GA-ABA interaction, we precociously germinated developing seeds *in vitro* with and without the use of plant growth regulators. The results from this research will provide further insights regarding hormonal regulation of seed development and *in vitro* precocious germination and thus contribute to the design of efficient and reproducible methodologies for accelerated breeding in legumes.

## Materials and Methods

This research was undertaken in the controlled plant growth facilities at the University of Western Australia, Perth (latitude: 31°58′49″ S; longitude: 115°49′7″ E). Pea (*P. sativum* L.) cultivars representing early (PBA Twilight), mid (PBA Pearl), and late (Kaspa) field flowering phenology were selected for this research. Plants were grown in two environments: Environment 1 (E1) optimized for rapid growth and development as per Croser et al. (2016): far-red enriched LED lighting–AP67, B series Valoya lights (Helsinki, Finland), and Environment 2 (E2) glasshouse under natural light conditions (February/March period) (**Table 1**).

**Table 1:** Environments and growth conditions used in this study.

Parameter	Environment 1	Environment 2
Temperature (day**/**night)	24°C/20°C	
Photoperiod	20 h	13–14 h
Light source	Far red-enriched LED*	natural light
Light intensity (µmol m^−2^ s^−1^)	300 (constant)	1,000 (midday)

*AP67, B series Valoya.

Seeds were sown in 0.4 L pots filled with steam pasteurized potting mix (UWA Plant Bio Mix–Richgro Garden Products Australia Pty Ltd). Plants were watered daily and fertilized weekly with a water-soluble N:P:K fertilizer (19:8.3:15.8) with micronutrients (Poly-feed, Greenhouse Grade; Haifa Chemical Ltd.) at a rate of 0.3 g per pot. Flowers were individually tagged at anthesis (when petals extended beyond the sepals).

### Effect of Growing Conditions on Seed Development and Its Effect on Precocious *in Vitro* Germination Ability

To study the effect of growing conditions on the rate of seed development, the fresh weight (mg seed^−1^) of seeds between 12 and 30 DAP produced in environments E1 and E2 was calculated. For this study, the mid flowering cultivar PBA Pearl was selected as a representative type, with a minimum of five seeds measured per developmental stage. Additionally, developing seeds around embryo physiology stage (between 14 and 22 DAP) produced in both environments were cultured *in vitro* to determine their ability for robust precocious germination as per Ribalta et al. (2017). Pods were surface-sterilized in 70% ethanol for 1 min, followed by 5 min in sodium hypochlorite (21 g/L), and three rinses in sterile deionized water. Pods were opened under sterile conditions, and 10 immature seeds, with and without integuments removed, were cultured per Petri dishes containing 20 mL MS medium (Murashige and Skoog, 1962) modified by the addition of 20% sucrose, 0.6% agar (Sigma, Type M), and pH 5.7. Germination percentage was recorded after 4 days of *in vitro* culture. Embryos were considered germinated when both radicle and shoot emergence was observed.

### Study of Hormone Profiles of Developing Seeds of Phenologically Diverse Pea Genotypes Produced in Different Environments

The aim of this experiment was to study the effect of plant growth conditions on the hormone profile of developing seeds from the end of morphogenesis to the beginning of embryo physiological maturity. Seeds of PBA Twilight, PBA Pearl, and Kaspa were harvested every 2 days in environment E1 from 10 to 22 DAP and in environment E2 from 14 to 22 DAP. For the hormone analysis, samples were formed from a pool of at least five seeds from different pods at each developmental stage. Seed integuments were removed, and samples stored at −80°C, before being freeze-dried at 20 µbar and 22°C using a VirTis^®^ , Bench Top^TM^ K series freeze dryer (Gardiner, NY, USA). The hormone extraction procedure was completed as per Lam et al. (2015). Quantification was performed by mass spectrometry with labeled internal standards. For auxin, details are provided by Lam et al. (2015) and Mc Adam et al. (2017) and for ABA by Mc Adam and Brodribb (2012). Gibberellins were analyzed without derivatization. For GA_1_, the transitions monitored for quantification were m/z 347 to 273 for endogenous GA_1_ and m/z 349 to 275 for the di-deuterated internal standard. For GA_20_, the transitions monitored were m/z 331 to 287 for endogenous and m/z 333 to 289 for the di-deuterated standard. The labeled GA internal standards were kindly provided by Prof. Lewis Mander of the Australian National University, Canberra. Hormone content levels were calculated based on DW (ng g^−1^).

### Role of Hormones on Precocious *in Vitro* Germination

To study the role of endogenous ABA as a preventer of precocious *in vitro* germination, seeds of the three phenologically diverse genotypes grown in E1 were collected at 18 DAP (embryo physiological maturity stage). Seeds were cultured as described above but with the addition of different ABA concentrations (0, 1, 2.5, 5, and 10 µM; A4906; Sigma-Aldrich, Australia). Seed coats were removed in all samples before culture. To determine the origin of endogenous ABA, seeds of the intermediate field flowering genotype PBA Pearl were also cultured with intact, nicked, and removed integuments on modified MS medium.

To study the promoting effect of GAs on precocious *in vitro* germination, seeds of the three genotypes grown in E1 were cultured at 12 and 14 DAP as previously described on modified MS medium with the addition of different concentrations of GA_3_ (0, 100, 125, and 150 µM; G7645; Sigma-Aldrich).

In all experiments, germination percentage was recorded after 4 days of *in vitro* culture for the ABA treatments and after 7 days for the GA_3_ treatments. Embryos were considered germinated when both radicle and shoot emergence was observed.

### Statistical Analysis

The effect of the environment on fresh weight of developing seeds was analyzed by Student *t* test (*P* ≤ 0.05). For the hormone profile analysis, data represent hormone content from a pool of at least five seeds from different plants, providing an average result of five individual plants. Data were analyzed by analysis of variance (*P* ≤ 0.05) to determine differences in hormone content between cultivars, seed developmental stages (DAP), and environments (n = 3). Two tests were run focusing on the period between the end of morphogenesis and initiation of seed dehydration in E1 (10–22 DAP) and on the period comprising the attainment of embryo physiological maturity in both environments (16–22 DAP). The environmental effect on seed hormone levels at the physiological maturity stage was analyzed by Student *t* test (*P* ≤ 0.05) by pooling hormone concentration data across genotypes, where no genotypic effect was observed (n = 3).

All *in vitro* precocious germination experiments were repeated at least three times with a minimum of 30 seeds per genotype and treatment. The experimental design was completely randomized, and the statistical analysis performed using χ^2^ test for homogeneity of the binomial distribution. A proportion test analysis was performed when significant differences between treatments were observed. Statistical tests were considered significant when *P* ≤ 0.05. All statistical analyses were performed using Rstudio software.

## Results

### Effect of Growing Conditions on Seed Growth and Development

**Figure 1:**
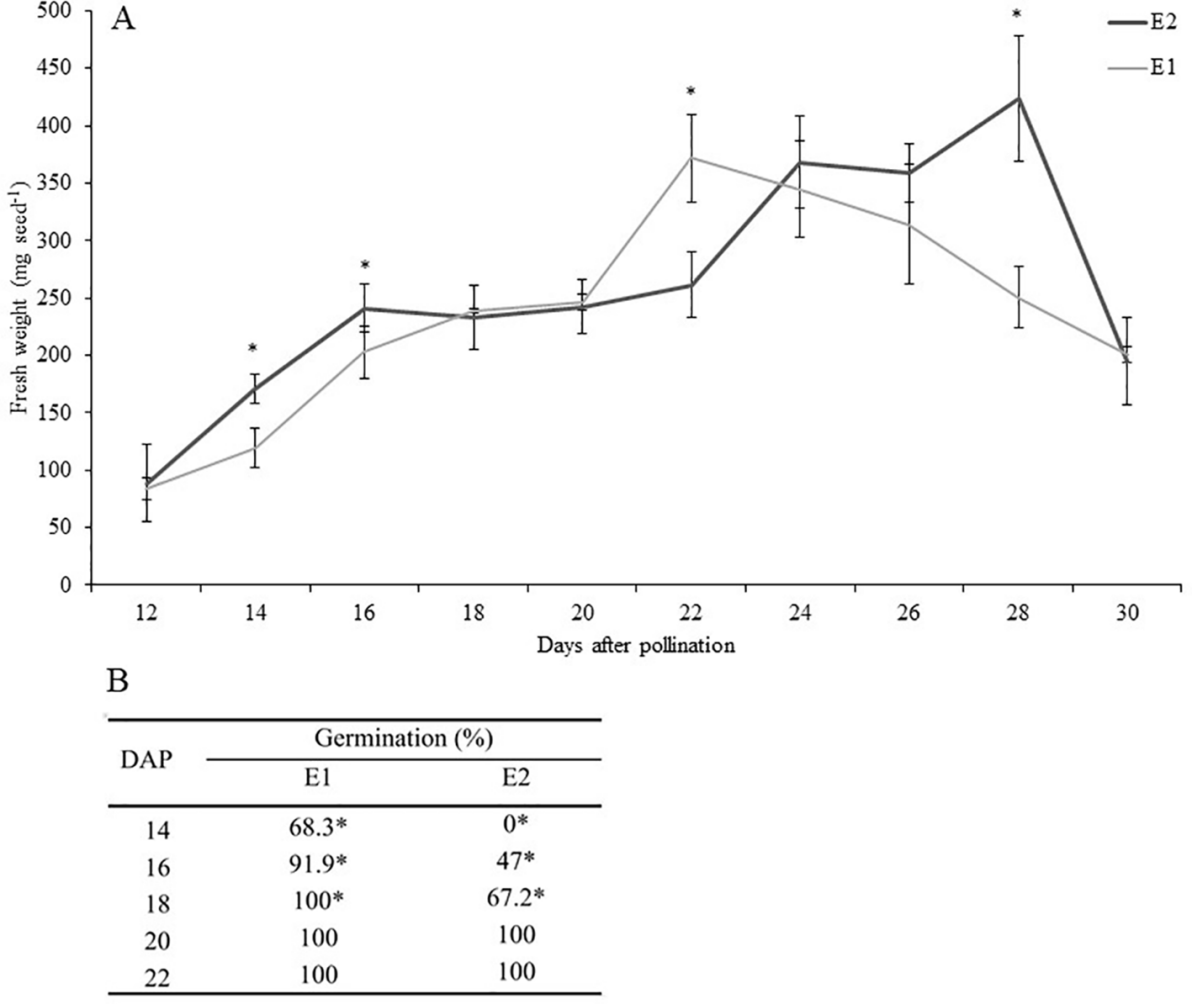
**(A)** Effect of growing conditions on fresh weight (mg seed^−1^) of developing seeds of PBA Pearl grown in environments E1 (controlled environment room) versus E2 (glasshouse). Data represent mean ± SE, n = 5. Analysis was performed by Student *t* test (*P* ≤ 0.05). **(B)** Precocious *in vitro* germination of PBA Pearl seeds 14 to 22 days after pollination (DAP) produced in environments E1 and E2. Seed coat was removed before culture. Results represent the percentage of germination 4 days after *in vitro* culture. Statistical analysis was performed using χ^2^ test for homogeneity of the binomial distribution (n = 30; *P* ≤ 0.05). Asterisks indicate significant differences between treatments.

The kinetics of development of PBA Pearl seeds in the environment optimized for rapid growth and development (E1) and the glasshouse environment (E2) are shown in **Figure 1A**. The largest difference in seed fresh weight occurred at 28 DAP (*P* ≤ 0.001), most likely attributable to seeds in E1 entering the desiccation phase at an earlier time point, as documented previously (Ribalta et al., 2017). This variation in development was also evidenced by the differential ability for robust *in vitro* germination of seeds harvested at equivalent time points from the two environments and without the use of plant growth regulators. In E1, *in vitro* germination levels greater than 91% were achieved by culturing immature seeds from 16 DAP, while in E2 similar levels of response were achieved only 4 days later (from 20 DAP, **Figure 1B**).

### Effect of Growing Conditions on the Hormone Profiles of Developing Seeds Around Embryo Physiological Maturity of Diverse Pea Genotypes

Experiments were undertaken to study the effect of plant growth conditions on the hormone profiles of developing seeds of phenologically diverse genotypes, from an approximate period between the end of embryo morphogenesis and the attainment of embryo physiological maturity stage, i.e. the period when the seed acquires the capacity for *in vitro* precocious germination. In our previous research, we demonstrated that embryo physiological maturity is achieved in pea under intensive conditions at 16 to 18 DAP (Ribalta et al., 2017). Therefore, for the hormone analysis in E1, we selected developing seeds between 10 and 22 DAP. To enable the comparison of the seed hormone profiles between environments, in E2 we selected immature seeds at equivalent developmental stages. The results from the experiment presented in the section above indicate a delay in seed development of approximately 2 to 4 days in the glasshouse environment (E2) compared to the optimized environment (E1). Based on these results, we estimate embryo physiological maturity is achieved in E2 at around 20 DAP, leading us to select immature seeds at 14 to 22 DAP for the hormone analysis.

*Auxins*. A strong environmental effect on endogenous 4-Cl-IAA and IAA content was observed when comparing the seed profiles during the period comprising the achievement of embryo physiological maturity under intensive (E1) and glasshouse (E2) conditions (16–22 DAP). This is clearly demonstrated by the statistical analysis shown in **Table S1D** (*P* < 0.001). Similar 4-Cl-IAA profile patterns were observed in E2 across genotypes with the highest concentrations, between 15,000 and 25,000 ng g DW^−1^, at 16 to 18 DAP. Across genotypes, the peak in 4-Cl-IAA content occurred much earlier in E1, typically at 10 to 12 DAP, so that by 16 to 18 DAP, seeds in E2 contained significantly higher hormone levels than E1 seeds (**Figures S1A, S2A, and S3A**). For example, at 16 DAP, in E2 the mean content of 4-Cl-IAA across genotypes was 17,190 ± 3,545 ng g DW^−1^ (n = 3), and in E1, 584 ± 218 ng g DW^−1^ (n = 3). This difference is significant at the *P* < 0.03 level (**Table S2**). In E1, the highest concentrations of IAA were observed at 10 DAP in the three genotypes and 4 to 8 days later in E2 (**Figures S1B, S2B, and S3B**; **Tables 2** and **S1**). Again, consistently higher concentrations of IAA were detected in seeds from E2 compared to those from E1 at 16 to 18 DAP (**Tables 2** and S1D). For example, at 16 DAP, the mean content of IAA across genotypes in E2 was 1,020 ± 229 ng g DW^−1^ (n = 3), while in E1 it was 34 ± 14 ng g DW^−1^ (n = 3), a difference significant at the *P* < 0.03 level (**Table S2**).

**Table 2:** Effect of growing conditions on hormone content (ng g DW^−^^1^) of developing seeds produced in environments E1 [10–22 days after pollination (DAP)] and E2 (14–22 DAP) for diverse pea genotypes.

Hormone	DAP	PBA Twilight		PBA Pearl		Kaspa	
		E1	E2	E1	E2	E1	E2
4-Cl-IAA	10	1,410.67 ± 571.03	—	12,917.77 ± 4,871.8	—	17,645.74	—
	12	300.1	—	19,227.6	—	36,381.6	—
	14	775.94	4,116.68	4,397.92 ± 644.72	3,951.1	1,386.61 ± 889.9	—
	16	1,012.96 ± 144.8	21,732.7	303.28 ± 26.22	19,634.8	436.95	10,204.00
	18	758.67 ± 68.84	24,818.1	338.93 ± 26.22	10,101.5	276.75 ± 6.18	15,982.00
	20	315.27 ± 15.53	2,185.59	500.18 ± 102.79	5,368.94	33.02 ± 8.37	1,081.69
	22	329.12 ± 29.21	619.35	66.92 ± 3.90	—	32.44 ± 3.65	455.15
IAA	10	2,447.46 ± 1,077.6	—	1,440.18 ± 66.8	—	859.38	—
	12	285.0	—	378.2	—	116.0	—
	14	1,098.49	808.64	697.33 ± 415.46	220.41	14.26 ± 6.98	—
	16	59.48 ± 1.43	699.34	30.48 ± 1.05	1,462.75	11.76	896.64
	18	17.60 ± 0.31	591.67	33.94 ± 1.31	162.49	5.64 ± 0.48	2,906.46
	20	5.72 ± 0.52	27.27	3.13 ± 0.83	76.73	2.34 ± 1.19	11.04
	22	5.11 ± 0.28	11.04	3.42 ± 0.34	—	4.87 ± 0.60	6.33
GA_20_	10	3,394.1 ± 3,075.7	—	81.81 ± 32.46	—	3,044.33	—
	12	2.46	—	3.69	—	543.12	—
	14	22.89	1,682.93	38.29 ± 34.81	4,742.38	3,854.68 ± 2,024.7	—
	16	1,908.40 ± 17.14	9,725.31	1,550.3 ± 33.86	7,290.94	2,322.96	5,392.42
	18	1,493.38 ± 19.77	16,991.8	963.39 ± 115.9	5,776.39	479.36 ± 24.39****	9,977.37
	20	1,182.52 ± 170.21	1,388.83	1,026.72 ± 345.49	3,068.67	37.54 ± 8.6	1,874.61
	22	2,732.65 ± 852.7	848.33	42.28 ± 6.18	—	9.57 ± 9.57	870.98
GA_1_	14	nd	57.41	nd	51.61	nd	—
	16	nd	4.82	nd	0	nd	703.47
	18	nd	5.01	nd	234.77	nd	373.16
	20	nd	0	nd	23.27	nd	0
	22	nd	39.32	nd	—	nd	0
ABA	10	688.11 ± 612.63	—	1,752.08 ± 1,373.2	—	7,213.1	—
	12	4,170.7	—	2,514.1	—	3,375.5	—
	14	3,246.52	355.01	4,997.41 ± 292.55	662.57	5,888.9 ± 4,082.4	—
	16	3,599.15 ± 18.01	1,080.19	1,936.34 ± 46.31	1,463.11	2,878.47	758.52
	18	1,659.13 ± 6.64	899.25	2,245.94 ± 6.44	674.07	4,555.8 ± 72.85	1,266.19
	20	5,596.92 ± 108.27	397.36	1,980.91 ± 617.1	397.58	3,421.36 ± 61.1	424.51
	22	2,387.55 ± 176.5	475.00	1,104.17 ± 114.36	—	2,898.67 ± 106.2	285.23

nd, not detected; —, not measured. Data represent mean hormone content from a pool of at least five seeds from different plants ± SE. Analysis of variance tests presented in **Table S1** show differences in seed hormone content between cultivars, developmental stages, and environments during the period between the end of morphogenesis and initiation of seed dehydration (10–22 DAP), and the period comprising the attainment of embryo physiological maturity in both environments (16–22 DAP; P ≤ 0.05; n = 3).

*Gibberellins*. Clear differences were observed in the GA_20_ profiles between environments. At the time points comprising the attainment of embryo physiological maturity in both environments (16–22 DAP), seeds in E2 contained significantly higher levels of GA_20_ than those in E1 (**Figures S1C, S2C, and S3C**; **Table S1D**). For example, at 16 DAP, seeds in E2 contained 7,469 ± 1,254 ng g DW^−1^ (n = 3) of GA_20_, while in E1 the level was 1,927 ± 223 ng g DW^−1^ (n = 3), a difference significant at the *P* < 0.03 level (**Table S2**). Furthermore, the well-defined peak in GA_20_ level observed in E2 was less apparent in E1. Also, when comparing GA_20_ concentrations between environments at the point of complete attainment of embryo physiological maturity in E1 (18 DAP), levels up to 20-fold higher were detected in E2 compared with E1. GA_1_ was only detected in E2 during the period studied (10–22 DAP; **Table 2**; **Figure S4**).

*Abscisic acid*. The statistical analysis in **Table S1D** showed a clear effect of controlled environment growth conditions on ABA concentrations between 16 and 22 DAP, with consistently lower levels detected in seeds in E2 compared to those grown in E1 (*P* < 0.01; **Figures S1D, S2D, and S3D**; **Table 2**). For example, across the three genotypes studied, at 16 DAP, the mean level of ABA in E2 was 1,100 ± 204 ng g DW^−1^ (n = 3), while in E1 it was 2,805 ± 482 ng g DW^−1^ (n = 3), a difference significant at the *P* < 0.03 level (**Table S2**). At the time point when full embryo physiological maturity is attained in E1 (18 DAP), ABA levels were again up to threefold higher under intensive conditions compared to the glasshouse.

### Role of Hormones on Precocious *in Vitro* Germination

To determine the origin of endogenous ABA, seeds of the cultivar PBA Pearl were cultured at the embryo physiological maturity stage (18 DAP) with intact, nicked, and removed integuments. The removal of the seed coat resulted in faster germination compared to the culture of intact or nicked seeds. After 4 days of culture, 100% germination was recorded in seeds with the seed coat removed, 70% with nicked seeds, and 9.1% with intact seeds (**Table S5**). All cultured seeds, independently of the treatment, germinated within 10 days of *in vitro* culture.

**Figure 2:**
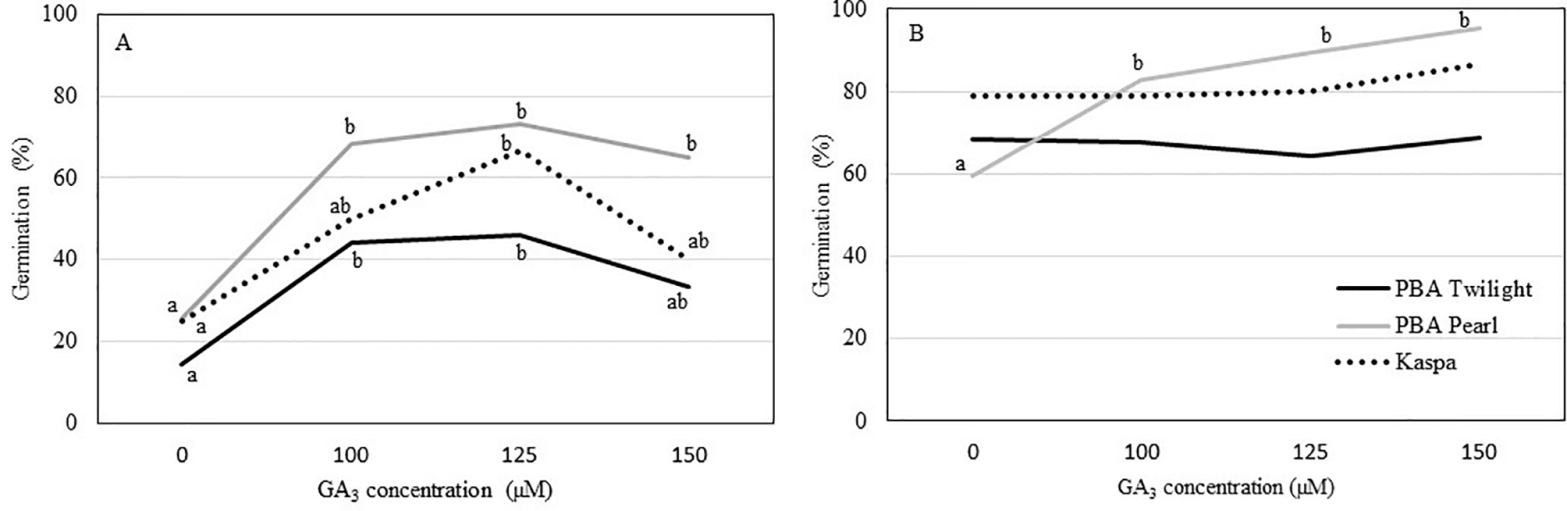
Effect of the addition of exogenous GA_3_ to the culture media on the percentage of *in vitro* germination of immature pea seeds **(A)** 12 days after pollination (DAP) and **(B)** 14 DAP from phenologically diverse genotypes. Statistical analysis was performed using χ^2^ test for homogeneity of the binomial distribution and proportional test (*P* ≤ 0.05; n = 30). Different letters indicate a difference at *P* < 0.05. Statistical data are presented in **Supplementary Table S3**.

Precocious *in vitro* germination of immature seeds at 12 DAP was enhanced in all three genotypes with the addition of GA_3_ to the culture medium. In general, growing seeds 12 DAP in media with GA_3_ concentrations up to 100 to 125 µM resulted in 2.5- to 3.5-fold increase in germination percentage compared to the control (*P* < 0.05; **Figure 2A**, **Table S3**). The addition of GA_3_ to the culture media had no effect on precocious germination of seeds 14 DAP in PBA Twilight and Kaspa. On the other hand, for PBA Pearl, exogenous GA_3_ at concentrations between 100 and 150 µM greatly enhanced the germination rate of 14-DAP seeds compared to the control (*P* < 0.001; **Figure 2B**, **Table S3**). Precocious *in vitro* germination rate was not significantly enhanced by increasing the concentration of GA_3_ to 125 and 150 µM at either 12 or 14 DAP.

**Figure 3:**
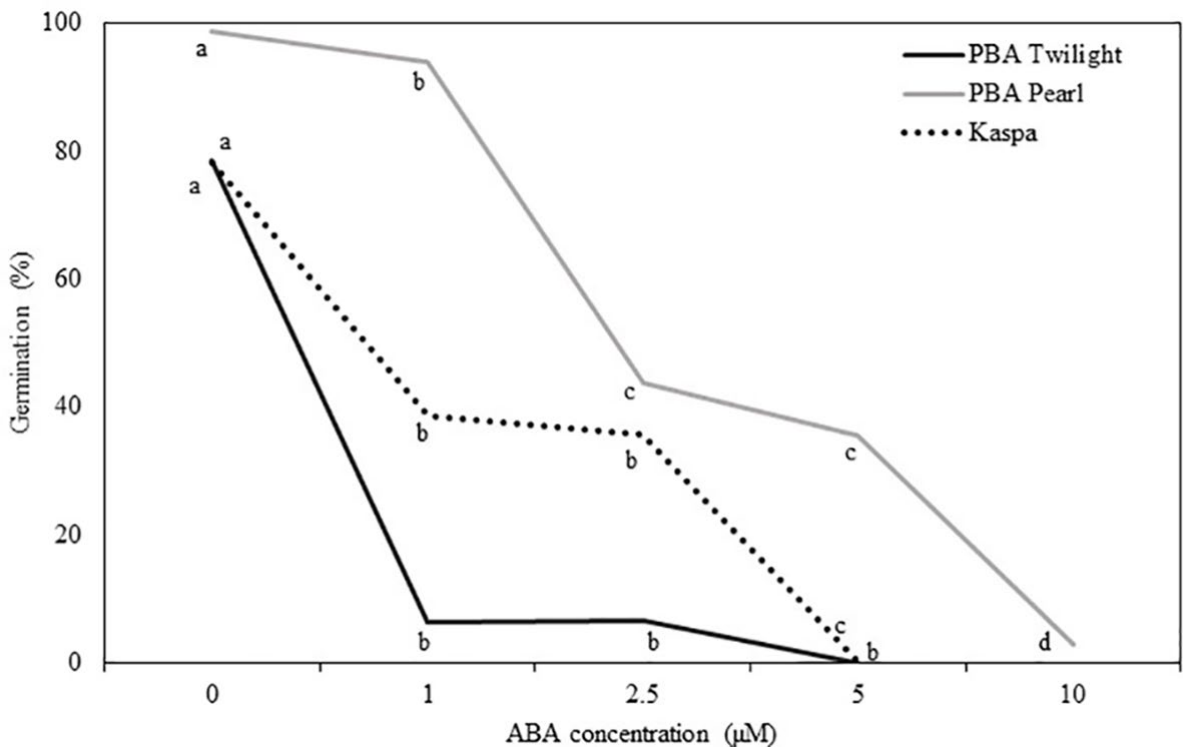
Effect of the addition of exogenous ABA to the culture media on *in vitro* germination of seeds at the embryo physiological maturity stage (18 days after pollination) from phenologically diverse pea genotypes. Statistical analysis was performed using χ^2^ test for homogeneity of the binomial distribution and proportional test (*P* ≤ 0.05; n = 30). Different letters indicate a difference at *P* < 0.05. Statistical data are presented in **Supplementary Table S4**.

Physiologically mature seeds (18 DAP) of the three genotypes tested showed different sensitivity to the addition of ABA to the culture medium (*P* < 0.001; **Figure 3**, **Table S4**). The addition of 1 µM of ABA reduced the germination rate in PBA Twilight to levels below 10%. Significantly higher levels of exogenous ABA were required to achieve similar levels of germination blockage in cultivars Kaspa (5 µM) and PBA Pearl (10 µM).

## Discussion

Hormones are known to regulate seed development, and their effect has been extensively studied in the model species *Arabidopsis* (Finkelstein, 2013; Binenbaum et al., 2018) and to some extent in *M. truncatula* (Ochatt, 2015). Despite this, little is known about the hormonal regulation of *in vitro* precocious seed germination in legumes (Weber et al., 2005; Ochatt, 2015; Croser et al., 2018). Here, we characterized and compared the hormone profiles of developing seeds harvested from three phenologically diverse pea genotypes from the end of morphogenesis to the attainment of embryo physiological maturity (10–22 DAP) and grown under two different controlled environments. The first environment (E1) was designed to promote rapid generation turnover for single seed descent (artificial light with a 20 h photoperiod). The second environment (E2) was a glasshouse used for normal plant growth and seed production activities (natural light and a photoperiod of 13–14 h). Growing plants under E1 conditions altered the seed hormone content by advancing the auxin, GA, and ABA profiles by 4 to 8 days compared to those of seeds grown under E2 conditions. We observed a synchronization of the IAA and 4-Cl-IAA profiles in E1 across the three genotypes. This was associated with an acceleration of the time to embryo physiological maturity by up to 4 days. In addition, we confirmed the antagonistic effect between exogenous ABA and GA on *in vitro* precocious seed germination.

The manipulation of key *in vivo* growth conditions, including photoperiod, light, and temperature, has enabled the substantial shortening of time to maturity in a broad range of species (reviewed by Croser et al., 2018). Our previous research in pea demonstrated sugar and moisture content of the developing seed varies in response to environmental conditions, and the resulting composition is linked to the achievement of embryo physiological maturity (Ribalta et al., 2017). By demonstrating that embryos did not efficiently germinate *in vitro* without exogenous hormones until physiological maturity was reached, we were able to propose sugar and moisture contents as a reliable indicator of readiness for precocious *in vitro* germination. In the present study, when comparing the kinetics of seed development between E1 and E2, the largest differences in seed fresh weight were detected at 28 DAP. This is in line with our previous report where we showed that seeds in the optimized environment reach the dehydration phase at an earlier time point (Ribalta et al., 2017). Nonsynchronous seed development across the two environments was further evidenced by the success rate of *in vitro* germination of seeds harvested at equivalent time points from the two environments and without the addition of exogenous plant growth regulators. For E1, *in vitro* germination levels greater than 91% were achieved by culturing immature seeds from 16 DAP, while for E2 similar levels of response were achieved from 20 DAP. The *in vitro* germination results informed the selection of seed developmental windows for hormone profiling. The focus of the hormone profile analyses was the study of the developmental period between the end of embryo morphogenesis and attainment of embryo physiological maturity, corresponding to the timeframe in which the seed acquires the capacity for *in vitro* precocious germination (Croser et al., 2016; Ribalta et al., 2017; Croser et al., 2018). Thus, under rapid generation turnover conditions, we undertook profile analysis on seeds between 10 and 22 DAP and under glasshouse conditions on seeds harvested 14 to 22 DAP.

Auxins are known to play a major role during the early stages of seed development. Evidence suggests an auxin-mediated promotion of GA synthesis is triggered by fertilization, driving early fruit growth (Dorcey et al., 2009). Recent evidence shows that in pea seeds auxins are also important during later stages of seed development for the determination of embryo structure and size, including starch accumulation (Locascio et al., 2014; Mc Adam et al., 2017). In the present research, clear differences across environments were detected at the time points comprising the period of attainment of embryo physiological maturity in both environments (16–22 DAP; *P* ≤ 0.001). Growing plants under conditions optimized for rapid generation turnover (E1) resulted in the acceleration of the 4-Cl-IAA profile by 6 to 8 days and of the IAA profile by 4 to 8 days compared to the glasshouse environment (E2). Depending on the genotype, we observed the highest 4-Cl-IAA and IAA levels at 10 to 12 DAP in E1 and at 16 to 20 DAP in E2, with a substantial lowering in concentration after that time point. Auxins have also been implicated in the onset and length of endoreduplication (Ochatt, 2015). Endoreduplication is a progressive phenomenon in storage accumulating organs during the transition between cell division and seed maturation phases (Kowles et al., 1990). This is concomitant with an increase in DNA synthesis and the accumulation of storage proteins, and there is a considerable agronomic interest in understanding the control of this phenomenon (Ochatt, 2011). In our research, the peak auxin concentrations observed in seeds produced in E1 (10–12 DAP) coincide with the peak endoreduplication observed in *M. truncatula* seeds (Ochatt, 2011; Atif et al., 2013). Our findings indicate that under E1 conditions, the end of morphogenesis and the concurrent initiation of embryo maturation and onset of endoreduplication occur between 10 and 12 DAP when the auxin peak is observed. Tivendale et al. (2012) provide further support for this association, reporting the relationship between decreasing concentrations of 4-Cl-IAA and IAA and the completion of seed development. Likewise, in E2, high auxin concentrations at later stages of seed development and for an extended period indicate that the cell division phase and endoreduplication are prolonged. Should this be the case, it is expected that the delay in seed development observed in E2 will translate into the production of seeds with higher number of cotyledonary cells (Ochatt, 2011; Ochatt, 2015), of a larger surface area (Atif et al., 2013), and probably coupled with a higher storage protein content (Gallardo et al., 2006; Verdier et al., 2013). Further studies in this area would be required to confirm these ideas.

Gibberellins have been recognized as regulators in numerous aspects of plant physiology, including embryo and seed development, induction of seed germination, root development, leaf expansion, stem elongation, and flowering (Salazar-Cerezo et al., 2018). Plants metabolize GAs through the early 13-hydroxylation pathway: GA_12_ → GA_53_ → GA_44_ → GA_19_ → GA_20_ → GA_1_ (Binenbaum et al., 2018), although there is evidence that in young pea seeds GA_1_ is produced from GA_4_ (MacKenzie-Hose et al., 1998). During the early stages of seed development, several peaks in the production of the bioactive gibberellin GA_1_ are observed that drive rapid seed coat and embryo growth. In pea, as the seeds enter into its maturation phase, a shift to the production of GA_20_ occurs with very low levels of bioactive GA detected in the embryo (Ozga et al., 2009). By the time seeds are dry, virtually all their GA_20_ has been converted to GA_29_ and then to GA_29_-catabolite (Ross et al., 1993). However, the biological significance of the later peaks of inactive GAs on the completion of seed development and subsequent germination is still not clear (Davidson et al., 2005; Ayele et al., 2006). In our experiment, seed GA levels were significantly affected by growth conditions (*P* ≤ 0.001). Using immature seeds around embryo physiological maturity, GA_1_ was only detected in seeds grown in E2, while its precursor, GA_20_, was detected in seeds from both environments. GA_20_ was observed at significantly lower levels (up to 20 times lower) at 18 DAP in Kaspa, and up to 8 days earlier in the profiles of seeds harvested in E1 compared to those from E2. The highest GA_20_ concentrations were detected between 10 and 16 DAP in E1 and 16 and 18 DAP in E2, with a sharp drop from this point of development. The reduced level of GA_20_ in E1, compared with E2, is one of the more dramatic effects on hormone content in this study. Mature dry pea seeds are known to contain very little GA_20_ (Ross et al., 1993), and the low level in E1 is consistent with the evidence from auxin levels indicating that these seeds are physiologically mature at an earlier stage than in E2. In our study, *in vitro* germination of seeds 12 and 14 DAP was 2.5- to 3-fold more successful with the addition of 125 µM GA_3_ to the culture medium. At 14 DAP, a clear improvement of *in vitro* precocious germination was observed only in PBA Pearl with +100 µM GA_3_. The enhancing effect of exogenous GA on precocious seed germination in pea is consistent with experiments showing promotion of α-amylase synthesis in germinating wheat seeds treated with GA_3_ (Hader et al., 2003; Kondhare et al., 2012).

Abscisic acid accumulates during seed maturation and in some species controls seed development and germination (Nakashima and Yamaguchi-Shinozaki, 2013). In *Arabidopsis*, reduced levels of ABA affect the induction of maturation genes leading to defective synthesis of storage proteins and anthocyanins, failed chlorophyll degradation, and causing precocious germination and intolerance to desiccation (Finkelstein, 2013). In developing seeds, ABA can be synthesized locally, originating from the embryo proper during seed maturation and showing peaks at the onset and at end of the maturation phase (Frey et al., 2004; Weber et al., 2005), or imported from the mother plant, through the seed coat (Quatrano et al., 1997). In the present study, as expected, ABA levels strongly fluctuated in response to the environmental conditions (*P* ≤ 0.001). In general, higher concentrations of ABA were detected in E1 compared to E2. Abscisic acid concentrations tended to decrease after reaching the highest levels at 10 to 14 DAP in E1 and 16 to 18 DAP in E2. To further understand the relevance of the ABA fluctuations observed in the hormone profiles to precocious seed germination, we applied exogenous ABA to seeds harvested at embryo physiological maturity (18 DAP) and cultured *in vitro*. Allowing the embryo to reach physiological maturity enables vigorous *in vitro* germination and faster seedling development with no requirement for plant growth regulators in the culture medium (Ribalta et al., 2017). In this experiment, ABA inhibited *in vitro* germination of physiologically mature embryos, with genotypic variations. A 5 µM ABA concentration was sufficient to completely block germination in PBA Twilight and Kaspa, while for PBA Pearl concentrations higher than 10 µM were required to achieve similar results. Additionally, to determine the origin of endogenous ABA, seeds of PBA Pearl were cultured at 18 DAP with intact, nicked, and removed integuments. The removal of the seed coat resulted in a 100% germination compared to 70% germination with nicked seeds and 9.1% germination with intact seeds. This suggests germination is slowed by mechanical impedance of the integuments rather than a hormonal barrier caused by the endogenous levels of ABA in the seed coat. This contrasts with results in *Arabidopsis* that indicate ABA produced in the seed coat affects precocious seed germination (Wang et al., 1998; Raz et al., 2001; Piskurewicz and Lopez-Molina, 2009). Also, the fact that all seeds from this experiment (intact seeds, nicked seeds, and seeds with coat removed) germinated within 10 days of *in vitro* culture highlights that seeds at 18 DAP are mature enough to complete germination.

A dynamic balance between ABA and GAs controls the progression of seed maturation to germination; therefore, there is ecological and commercial value in understanding this physiological regulation (Weber et al., 2005; Rodriguez-Gacio et al., 2009; Liu et al., 2010; Ochatt, 2015). Abscisic acid levels increase during the late phase of seed maturation and are maintained until germination, while GA concentrations remain relatively low during this period until seed imbibition (Ogawa et al., 2003; Locascio et al., 2014). The *FUS3* gene plays an essential role in the coordination of ABA: GA levels during the late stages of seed development and germination. The *FUS3* gene regulates ABA and GA synthesis, and these two hormones in turn determine the stability of the FUS3 protein (Gazzarrini et al., 2004). Gibberellin is negatively regulated by *FUS3*, while ABA is a positive regulator of many *FUS3*-regulated embryonic functions including storage reserve accumulation, desiccation tolerance, and dormancy (Keith et al., 1994; Bäumlein et al., 1994; Leung and Giraudat, 1998; Gazzarrini et al., 2004). Hence, the ABA : GAs ratio is crucial for the completion of seed maturation and the initiation of germination (Liu et al., 2010; Locascio et al., 2014; Ochatt, 2015). As previously indicated, in this study we confirmed the antagonistic effect between ABA and GA on pea seed germination through the *in vitro* culture of seeds at the embryo physiological maturity (18 DAP) with the addition of exogenous hormones to the media. The ratio of ABA to GA was proposed as an indicator of embryo maturation for *in vitro* culture studies in legumes (Slater et al., 2013). Seed endogenous ABA concentrations are known to increase as the seed matures; however, being a stress-response hormone, ABA levels also fluctuate during the day in response to environmental signals. Therefore, the ratio of ABA to GA is not a reliable indicator of embryo physiological maturity when growing plants under conditions for rapid generation turnover. On the other hand, the earlier peak in auxin and GA production in E1 compared with E2 is likely to contribute to the earlier attainment of physiologically maturity and earlier competence to germinate in E1, since auxin-deficient seeds do not develop normally, and their germination rate is low (Mc Adam et al., 2017). This suggests auxin and GA profiles can act as reliable indicators of the end of morphogenesis and the initiation of seed maturation.

Developmental and environmental signals (such as water potential, temperature, and light quality) influence endogenous hormone levels in developing seeds and the complex signaling connections between hormones and sugars, which ultimately control seed size, starch and protein accumulation, dormancy, and germination (Piskurewicz et al., 2009; Rodriguez-Gacio et al., 2009; Locascio et al., 2014). In the present study, we provide new information regarding the influence of growing conditions on the progress of seed development and maturation and on endogenous hormone accumulation across diverse genotypes of the model legume species pea. These results will provide further insights into the hormonal regulation of legume seed development and *in vitro* precocious germination and contribute to the design of efficient and reproducible biotechnological tools contributing to genetic gain.

## Data Availability Statement

The raw data supporting the conclusions of this manuscript will be made available by the authors, without undue reservation, to any qualified researcher.

## Author Contributions

FR, MP-N, JC, SO, and JR conducted experimental design, data analysis/interpretation, and manuscript writing. KE and FR conducted *in vitro* experiments and data collection. MP-N and JR conducted hormone analysis.

## Funding

The UWA authors acknowledge funding provided by the Grains Research and Development Corporation [UWA00175].

## Conflict of Interest

The authors declare that the research was conducted in the absence of any commercial or financial relationships that could be construed as a potential conflict of interest.
