# Supplementary material for: Expression Patterns of Key Hormones Related to Pea (Pisum sativum L.) Embryo Physiological Maturity Shift in Response to Accelerated Growth Conditions
Source: Front Plant Sci. 2019 Sep 27;10:1154. doi: 10.3389/fpls.2019.01154 (PMC6776635; doi:10.3389/fpls.2019.01154)
Supplement: Supplementary file 1 [file Table_1.docx]

Supplementary Material

# Supplementary Figures


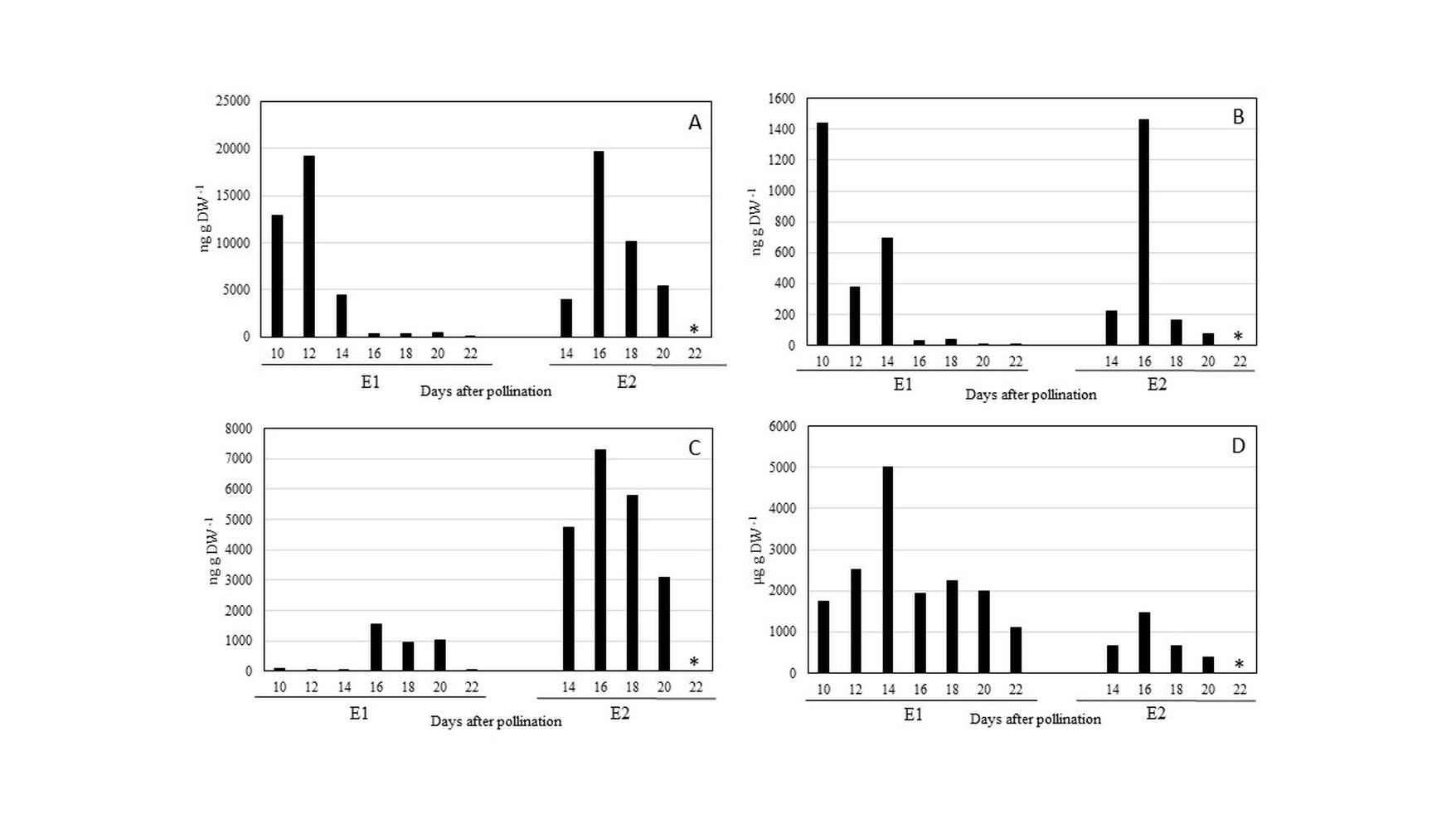


**Supplementary Figure 1**. Effect of growing conditions on endogenous hormone content (ng g DW^-1^) of developing seeds of cv. PBA Pearl produced in E1 (environment optimized for rapid generation turnover; 10-22 days after pollination) and E2 (glasshouse; 14-22 days after pollination). (**A)** 4-Cl-IAA; (**B)** IAA, (**C)** GA_20_ and (**D)** ABA. * = not measured. Data represent hormone content from a pool of at least five seeds from different plants. A multivariate test (P ≤ 0.05) was performed to determine the differences between cultivars and seed developmental stages (n=3). Statistical data are presented in Supplementary Tables S1 and S2.


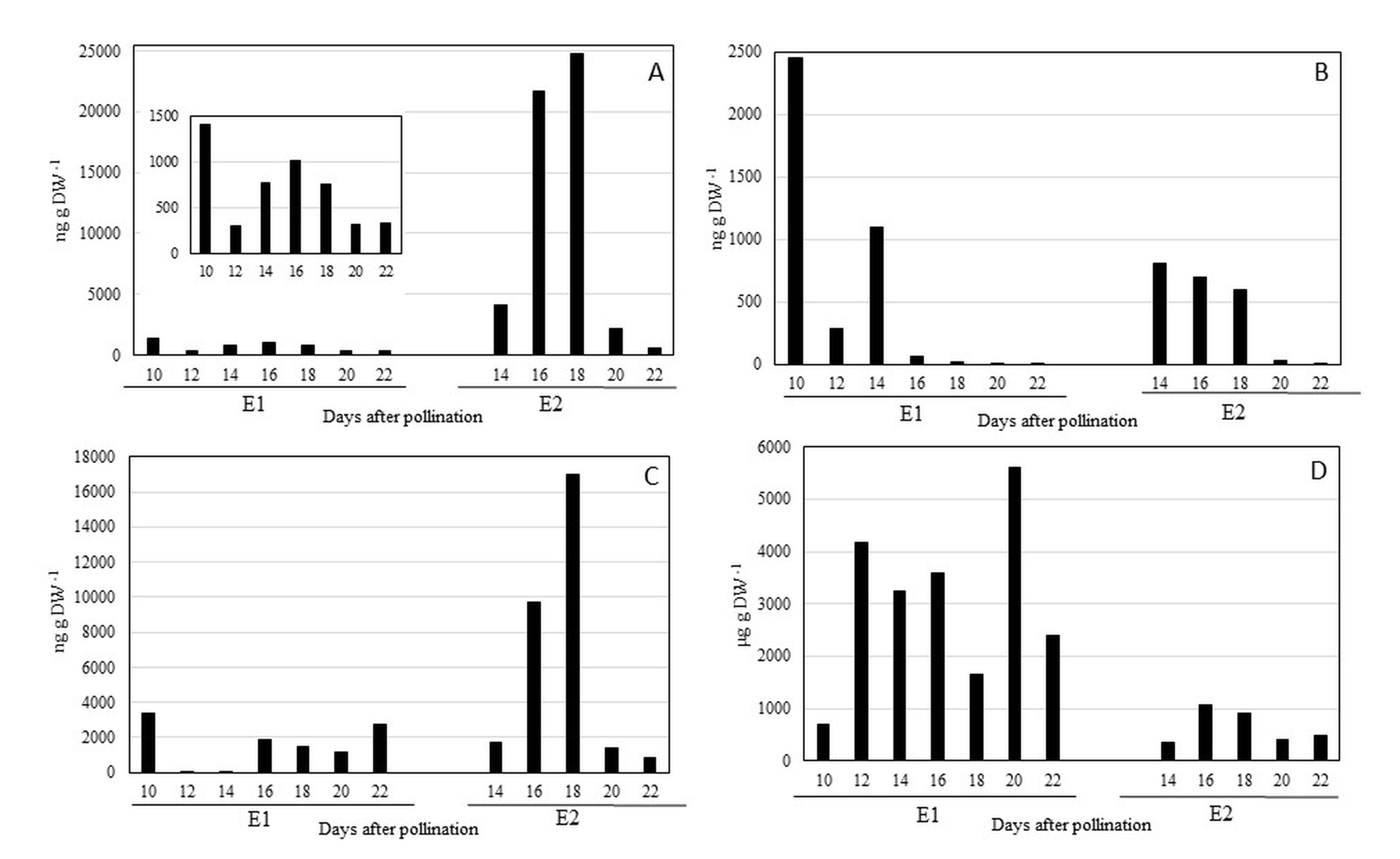


**Supplementary Figure 2**. Effect of growing conditions on endogenous hormone content (ng g DW^-1^) of developing seeds of cv. PBA Twilight produced in E1 (environment optimized for rapid generation turnover; 10-22 days after pollination) and E2 (glasshouse; 14-22 days after pollination,). (**A**) 4-Cl-IAA, insert shows in more detail the 4Cl-IAA levels detected in E1; (**B**) IAA, (**C)** GA_20_ and (**D**) ABA. Data represent hormone content from a pool of at least five seeds from different plants. A multivariate test (P<0.05) was performed to determine the differences between cultivars and seed developmental stages (n=3). Statistical data are presented in Supplementary Tables S1 and S2.

**
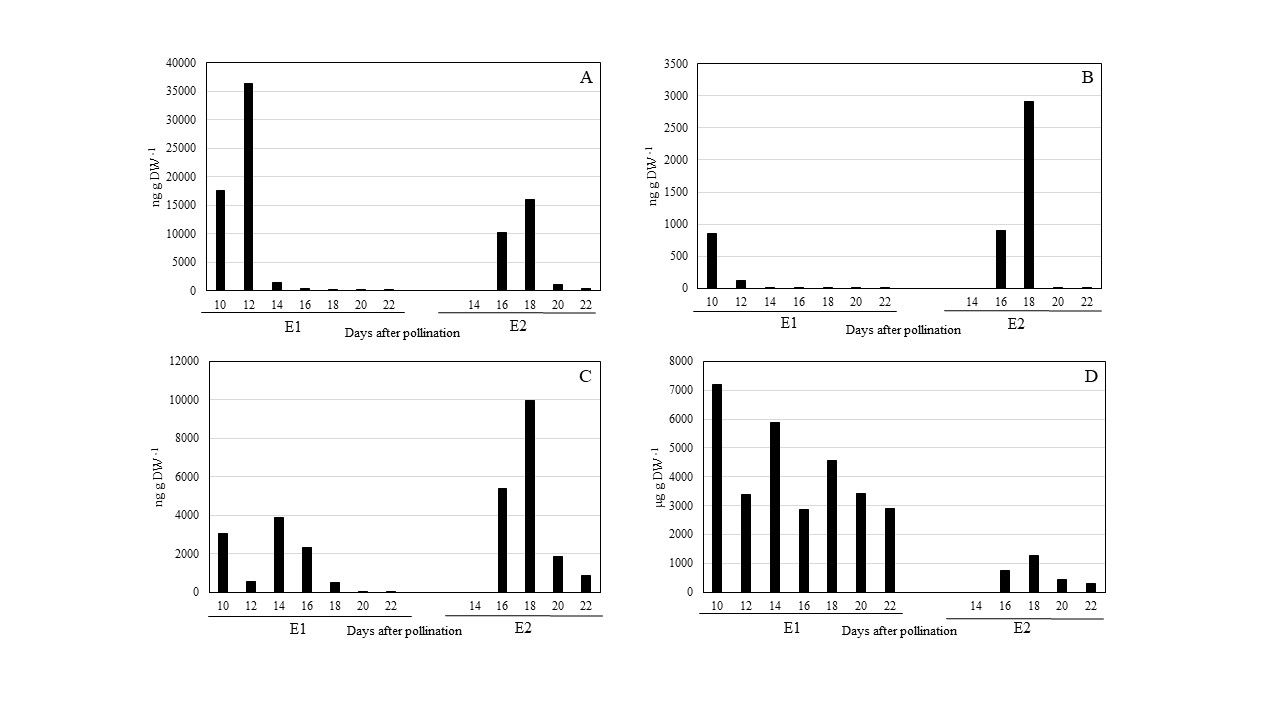
Supplementary Figure 3**. Effect of growing conditions on endogenous hormone content (ng g DW^-1^) of developing seeds of cv. Kaspa produced in E1 (environment optimized for rapid generation turnover; 10-22 days after pollination) and E2 (glasshouse; 14-22 days after pollination). (**A)** 4-Cl-IAA; (**B**) IAA, (**C)** GA_20_ and (**D)** ABA. Data represent hormone content from a pool of at least five seeds from different plants. A multivariate test (P < 0.05) was performed to determine the differences between cultivars, seed developmental stages and environments (n=3). Statistical data are presented in Supplementary Tables S1 and S2.

°

°

°

°

*

*

**Supplementary Figure 4.** GA_1_ content (ng g DW^-1^) of developing seeds 14-22 days after pollination produced in environment E2 for diverse pea genotypes. ° = not detected; * = not measured. Data represent hormone content from a pool of at least five seeds from different plants.

**Supplementary Tables**

**Supplementary Table 1.** ANOVA of seed hormone level data during (A and B) the period between the end of morphogenesis and initiation of seed dehydration in E1 (10-22 days after pollination, DAP), and (C and D) the period comprising the attainment of embryo physiological maturity in both E1 and E2 environments (16-22 DAP) (n=3).

1. ANOVA by developmental stage – p-values (10-22 DAP)

|  | PBA Twilight | PBA Pearl | Kaspa |
| --- | --- | --- | --- |
| 4-Cl-IAA | 0.01292 | 0.001147 | 0.01299 |
| IAA | 0.004535 | 0.0004765 | 0.02116 |
| GA_20_ | 0.9575 | 0.5314 | 0.01317 |
| ABA | 0.2646 | 0.1819 | 0.09513 |

1. ANOVA by genotype – p-values (10-22 DAP)

| 4-Cl-IAA | 0.01712 |
| --- | --- |
| IAA | 0.3551 |
| GA_20_ | 0.1998 |
| ABA | 0.05999 |

1. Environment 1– p-values (16-22 DAP)

|  | By genotype | By DAP |
| --- | --- | --- |
| 4-Cl-IAA | 8.053 e-07 | 1.637 e-06 |
| IAA | 0.0003286 | 7.171 e-08 |
| GA_20_ | 9.138 e-05 | 0.068209 |
| ABA | 0.003347 | 0.20689 |

1. Environmental effect – p-values (16-22 DAP)

| 4-Cl-IAA | 5.850 e-11 |
| --- | --- |
| IAA | 0.0006448 |
| GA_20_ | 1.491 e-06 |
| ABA | 1.087 e-06 |

**Supplementary Table 2.** Student’s t-test of the environmental effect (E1 vs E2) on seed hormone content 16 days after pollination (time-point of the onset of embryo physiological maturity in the intensive environment E1). As no significant differences between genotypes were observed during this developmental period, hormone concentration data were pooled across genotypes (n=3). Only p-values are shown to highlight differences between environments**.**

| 4-Cl-IAA | 0.020199 |
| --- | --- |
| IAA | 0.026049 |
| GA_20_ | 0.028161 |
| ABA | 0.015542 |

**Supplementary Table 3. (A)** χ^2^ test for homogeneity of the binomial distribution on *in vitro* germination percentages (%) of seeds 12 and 14 days after pollination (DAP) after seven days of culture in GA_3_ containing media (n =30). Asterisks indicate where significant differences between treatments were observed (P<0.05). **(B)** Proportion test analysis (prop.test) performed when significant differences between treatments were observed to establish treatments different among them. Only p-values are shown to highlight differences between treatments.

**(A)**

| Genotype/ seed developmental stage | GA_3_ concentration (µM) | | | |
| --- | --- | --- | --- | --- |
|  | 0 | 100 | 125 | 150 |
| PBA Twilight 12 DAP* | 12.9% | 42.85% | 45.5% | 33.33% |
| X-squared = 8.2184, df = 3, p-value = 0.04171 | | | | |
| PBA Twilight 14 DAP | 68.57% | 67.74% | 64.51% | 68.75% |
| X-squared = 0.15271, df = 3, p-value = 0.9848 | | | | |
| PBA Pearl 12 DAP* | 25% | 68.29% | 72.97% | 65% |
| X-squared = 35.747, df = 3, p-value = 8.49e-08 | | | | |
| PBA Pearl 14 DAP* | 59.66% | 82.89% | 89.58% | 95.12% |
| X-squared = 32.518, df = 3, p-value = 4.07e-07 | | | | |
| Kaspa 12 DAP* | 25% | 50% | 66.67% | 40% |
| X-squared = 11.992, df = 3, p-value = 0.00712 | | | | |
| Kaspa 14 DAP | 78.95% | 78.95% | 80% | 86.67% |
| X-squared = 0.42545, df = 3, p-value = 0.9349 | | | | |

**(B)**

| Genotype/ seed developmental stage | GA_3_ concentration (µM) | 0 | 100 | 125 | 150 |
| --- | --- | --- | --- | --- | --- |
| PBA Twilight 12 DAP | 0 | 1 | 0.0339 | 0.01968 | 0.1025 |
|  | 100 |  | 1 | 1 | 0.6763 |
|  | 125 |  |  | 1 | 0.5332 |
|  | 150 |  |  |  | 1 |
| PBA Pearl 12 DAP | 0 | 1 | 1.3e-05 | 3.06e-06 | 6.28e-05 |
|  | 100 |  | 1 | 0.8383 | 0.9374 |
|  | 125 |  |  | 1 | 0.6111 |
|  | 150 |  |  |  | 1 |
| PBA Pearl 14 DAP | 0 | 1 | 0.001138 | 0.000349 | 5.60e-05 |
|  | 100 |  | 1 | 0.4424 | 0.1101 |
|  | 125 |  |  | 1 | 0.567 |
|  | 150 |  |  |  | 1 |
| Kaspa  12 DAP | 0 | 1 | 0.07616 | 0.001808 | 0.3218 |
|  | 100 |  | 1 | 0.2776 | 0.6038 |
|  | 125 |  |  | 1 | 0.06179 |
|  | 150 |  |  |  | 1 |

**Supplementary Table 4. (A)** χ^2^ test for homogeneity of the binomial distribution on *in vitro* germination percentages (%). Seeds at the stage when embryo physiological maturity is fully attained (18 DAP) were cultured in media containing different concentrations of ABA (n= 30). Germination percentages were recorded four days after culture. Asterisks indicate where significant differences between treatments were observed (P<0.05). **(B)** Proportion test analysis (prop.test) performed when significant differences between treatments were observed to establish the treatments that were different among them. Only p-values are shown to highlight differences between ABA treatments.

**(A)**

| Genotype | ABA concentration (µM) | | | | |
| --- | --- | --- | --- | --- | --- |
|  | 0 | 1 | 2.5 | 5 | 10 |
| PBA Twilight* | 80% | 6.65% | 6.67% | 0% | na |
| X-squared = 82.543, df = 3, p-value = 2.2e-16 | | | | | |
| PBA Pearl* | 98.75% | 93.93% | 43.75% | 35.56% | 2.94% |
| X-squared = 3129.5776, df = 3, p-value = 4.794405e-27 | | | | | |
| Kaspa* | 82.5% | 38.5% | 35.7% | 0% | na |
| X-squared = 47.28526, df = 3, p-value = 3.022377e-10 | | | | | |

**(B)**

| Genotype | ABA concentration (µM) | 0 | 1 | 2.5 | 5 | 10 |
| --- | --- | --- | --- | --- | --- | --- |
| PBA Twilight | 0 | 1 | 1.29e-09 | 2.13e-09 | 8.97e-11 | na |
|  | 1 |  | 1 | 1 | 0.5018 | na |
|  | 2.5 |  |  | 1 | 0.487 | na |
|  | 5 |  |  |  | 1 | na |
|  | 10 | na | na | na | na | na |
| PBA Pearl | 0 | 1 | 4.22e-01 | 1.73e-11 | 1.14e-14 | 2.20e-16 |
|  | 1 |  | 1 | 3.88e-05 | 6.64e-07 | 5.57e-13 |
|  | 2.5 |  |  | 1 | 0.6244 | 0.000252 |
|  | 5 |  |  |  | 1 | 0.001299 |
|  | 10 |  |  |  |  | 1 |
| Kaspa | 0 | 1 | 6.63e-04 | 2.31e-04 | 1.10e-10 | na |
|  | 1 |  | 1 | 1 | 0.00102 | na |
|  | 2.5 |  |  | 1 | 0.001688 | na |
|  | 5 |  |  |  | 1 | na |
|  | 10 | na | na | na | na | na |

**Supplementary Table S5.** Effect of seed coat nicking and removal on *in vitro* germination of seeds at the stage when embryo physiological maturity is fully attained (18 DAP). Results represent the percentage of germination four days after *in vitro* culture of the mid flowering genotype PBA Pearl. Statistical analysis was performed using χ^2^ test for homogeneity of the binomial distribution (n= 30; P < 0.05). Asterisks indicate where significant differences between treatments were observed.

| Treatment | Germination (%) |
| --- | --- |
| Seed coat removed | 100 |
| ^1^Nicked seed coat | 70* |
| ^1^Intact seed coat | 9.1* |

^1^100% germination was achieved 10 days after culture.
